# Does Retirement Make People Happier?-Evidence From China

**DOI:** 10.3389/fpubh.2022.874500

**Published:** 2022-06-17

**Authors:** Anqi Zhang, Yi Zhang, Yiwen Tao

**Affiliations:** ^1^School of Public Economics and Administration, Shanghai University of Finance and Economics, Shanghai, China; ^2^School of Public Administration, Zhongnan University of Economics and Law, Wuhan, China

**Keywords:** retirement, RD, happiness, policy, role

## Abstract

To investigate whether people's happiness will increase after retirement, this paper empirically investigates 2012, 2015, and 2017 China General Social Survey (CGSS) data using Ordinary Least Squares, Binary logit, and Fuzzy regression discontinuity Design. The results all show that retirement will significantly increase the happiness of men in urban China. The paper also validates these findings by testing the continuity of the reference and covariates at the cut–off point, changing the model settings, and using a more rigorous sample classification method. In addition, the article further analyzes the heterogeneity of the study and finds that retirement brings more happiness to those who have a college degree or less and have multiple children. The better the health status, the smaller the effect of retirement on happiness. The more social interactions, the smaller the effect of retirement on happiness. The policy implication of this paper is that when implementing a delayed retirement policy, special care should be taken for groups with greater welfare impairment, and it needs to be introduced together with other supporting measures to reduce people's worries. This paper analyzes the relationship between retirement and happiness in China and makes suggestions for the implementation of a delayed retirement policy, enriching the theoretical and empirical work on the effects of retirement on people's happiness and contributing to the world's response to aging and welfare policies for the older person.

## Introduction

With the generational change in population, the baby boomers born in the 1950s and 1960s in China will gradually exit the labor market and enter retirement. Retirement is an important turning point in life, the end of labor, and the beginning of retirement. It implies changes in work, life, and psychology, and therefore, changes in happiness before and after retirement have attracted the attention of more and more scholars.

On the one hand, compared with before retirement, the elderly after retirement may reduce unhealthy, high–risk, harmful, and stressful jobs, and have more time to participate in entertainment–oriented activities to promote brain activities, enhance relationships with their families and protect mental health, thus increasing the happiness of the older person (1–4), especially for voluntary retirees (5, 6). On the other hand, retirement changes individuals' daily routines, physical and mental activities, reduces income levels, and at the same time brings individuals a sense of loneliness, low value, thus reducing people's happiness (7, 8).

Research on the impact of retirement on the happiness of the older person in China, the extent of the impact, and the channels of the impact will provide important inspiration for China's upcoming retirement policy and will also provide important implications for the world's response to aging and the improvement of the welfare of the older person. At the same time, it has enriched the theoretical and empirical work on the impact of retirement on happiness. In particular, it should be noted that the happiness studied in this paper is a proxy for subjective well–being in a broad sense. The rural residents' pension insurance system started late and the level of protection is very low. Basically, after retirement, they still engage in agricultural production, and retirement or not has no impact on their lives. Since female workers are mostly affected by their spouses' work status and family conditions, their retirement situation is more complicated. and men in China's urban areas are more compliant with the work and retirement system. Therefore, in this paper, only the impact of retirement on the happiness of urban men is studied.

At present, empirical research on the impact of retirement on happiness has mixed conclusions. Some research found that retirement had a negative effect on happiness, while others believed that the effect was positive. Early research on the relationship between retirement and happiness is derived from psychology, and mainly describes the relationship between retirement and happiness. These studies concluded that retirement is associated with lower life satisfaction, depression, and lower happiness (9, 10). Some scholars have explained the reason for the decline in happiness after retirement. First, the decline in individual happiness after retirement is mainly due to the reduction of personal income and other key resources. Second, retirement will deprive individuals of their social role in the social division of labor and have an adverse impact on their sense of social identity, thus easily triggering negative emotions, and reducing the quality of life (11, 12); from the perspective of family relations, concluded that no matter who the spouse retired first, the right to speak in the family would be reduced, which in turn would reduce their satisfaction with life (13). This indicates that retirement will reduce the happiness of married people. Some Chinese scholars have found that environmental information, regulation, and governance have a significant impact on happiness (14–18).

Some scholars have argued that retirement will significantly improve the happiness of residents. For example, Some research studied data from multiple US public opinion surveys and found that retirement will make the older person feel more satisfied with their quality of life, and the impact on men is significantly greater than that on women (19). Jokela et al. further supported the above views, and believed that retirement will increase the individual's leisure time and reduce their pressure from work and competition, and therefore help increase their happiness (20); some studies have also confirmed that retirement will significantly increase people's happiness (21).

At the same time, some scholars argue that the effect of retirement on happiness is not significant. The empirical research of Beck shows that retirement has no obvious effect on changes in happiness. This is mainly due to the advantages and disadvantages of retirement for individuals, and it is a neutral event in the life course (7). According to role theory, retirement may have a negative or positive effect on the happiness of an individual, it depends on the individual's perspective on the role, so if we only focus on the ultimate effect of retirement on life satisfaction, it is difficult to draw significant conclusions.

Existing research discusses the negative or positive effects of retirement on happiness, but it lacks effective treatment of endogeneity and cannot get rid of the mutual causality between retirement and happiness. Researchers tried to clarify the relationship between retirement and happiness through pension rules, but the results are still uncertain (22). At the same time, the existing research lacks the corresponding mechanism analysis of the impact of retirement on happiness. Considering the particularities of China's pension system, such as involuntary retirement, and the internal validity issues in the study of the Chinese sample, whether the empirical findings of other countries in the world are applicable to China requires further empirical analysis of China's samples with more precise methods.

Based on the deficiencies of previous research, such as less attention to the Chinese sample, the internal validity of the research on the Chinese sample, and the insufficient research on the internal mechanism, this paper uses the Chinese urban household registration population in the CGSS data in 2012, 2015 and 2017 as the research object, and adopts the fuzzy regression discontinuity design to more accurately identify the causal relationship of the impact of retirement on happiness and examine the group differences in the impact of retirement on happiness and the ways in which retirement affects happiness. This article enriches the empirical research on the impact of retirement on happiness and provides empirical material for further theoretical work on the impact of retirement on happiness. In addition, the research has important inspiration for China's upcoming retirement policy and is of great significance to the world's policy formulation in response to aging and improving the welfare of the older person. In particular, it provides a certain reference significance for the formulation of retirement policies in developing countries. At the same time, it has enriched the theoretical and empirical work on the impact of retirement on happiness.

Our research has found that reaching the national pension granting age will cause a large proportion of Chinese people to retire. Retirement greatly improves personal happiness, and its impact is significant and robust. Second, we found that retirement brings more happiness to those who have a college degree or less and have multiple children. The better the health status, the smaller the effect of retirement on happiness. The more social interactions, the smaller the effect of retirement on happiness. The method used in this paper provides a clear framework for the research on how retirement affects happiness.

The structure of the article is as follows: The second part is the theoretical basis, and the third part mainly includes the data sources, the setting of the variables and their basis, descriptive statistics, and its analysis; The fourth part is the empirical results, it mainly elaborates the regression results of the three models of Ordinary Least Squares (OLS), binary Logit and Fuzzy Breakpoint Regression (FRD); The fifth part is a further discussion on the continuity of the reference variable and the covariate at the breakpoint, the test of the regression results of different sample intervals, and the analysis of heterogeneity. The sixth part is the conclusion and policy implication.

## Theoretical Basis

Role theory suggests that society is made up of people with different statuses, positions, occupations, and personalities. When a person expresses views and opinions consistent with his or her personality, performs the duties and work of a position, or fulfills the rights and obligations of his or her social status, we can assume that the person is playing a certain role. Each person is playing multiple roles at the same time.

Retirement means that the older person no longer plays the role in the frontline job position and returns to the family and the life which is unfamiliar and far away from work to play the role that society and life require him to play. In this process, role changes and conflicts will occur. Work is a source of self–esteem and self–identity and plays a central role in the lives of adults. Retirement ends this job role, and the retired individual will suffer psychologically because he/she can no longer see himself as a productive and contributing member of society. The theory holds that leisure roles during retirement cannot replace work roles as a source of self–esteem (2).

Some scholars argue that individuals play multiple roles, such as family roles and friend roles. These roles are continuous, and individuals tend to maintain their original state after retirement. Retirement will not change people much (23, 24). The job role is not necessarily their core role. Retirement can provide an opportunity for them to spend more time on the important roles of friends and family, and the continuity of these roles after retirement will prevent overall negative consequences of retirement. The author further pointed out that retirement can alleviate the work pressure caused by performance requirements, which can actually improve mental health.

The theory of continuing socialization of old age believes that in old age, individuals still need to produce new values through continuous learning, such as proficient use of basic life skills, consciously abiding by social behavior norms, and gradually adjusting social relations; while constantly learning new knowledge, new skills, taking on new social roles and establishing new social relationships, in order to adapt to society and achieve self–improvement (25).

**Figure 1:**
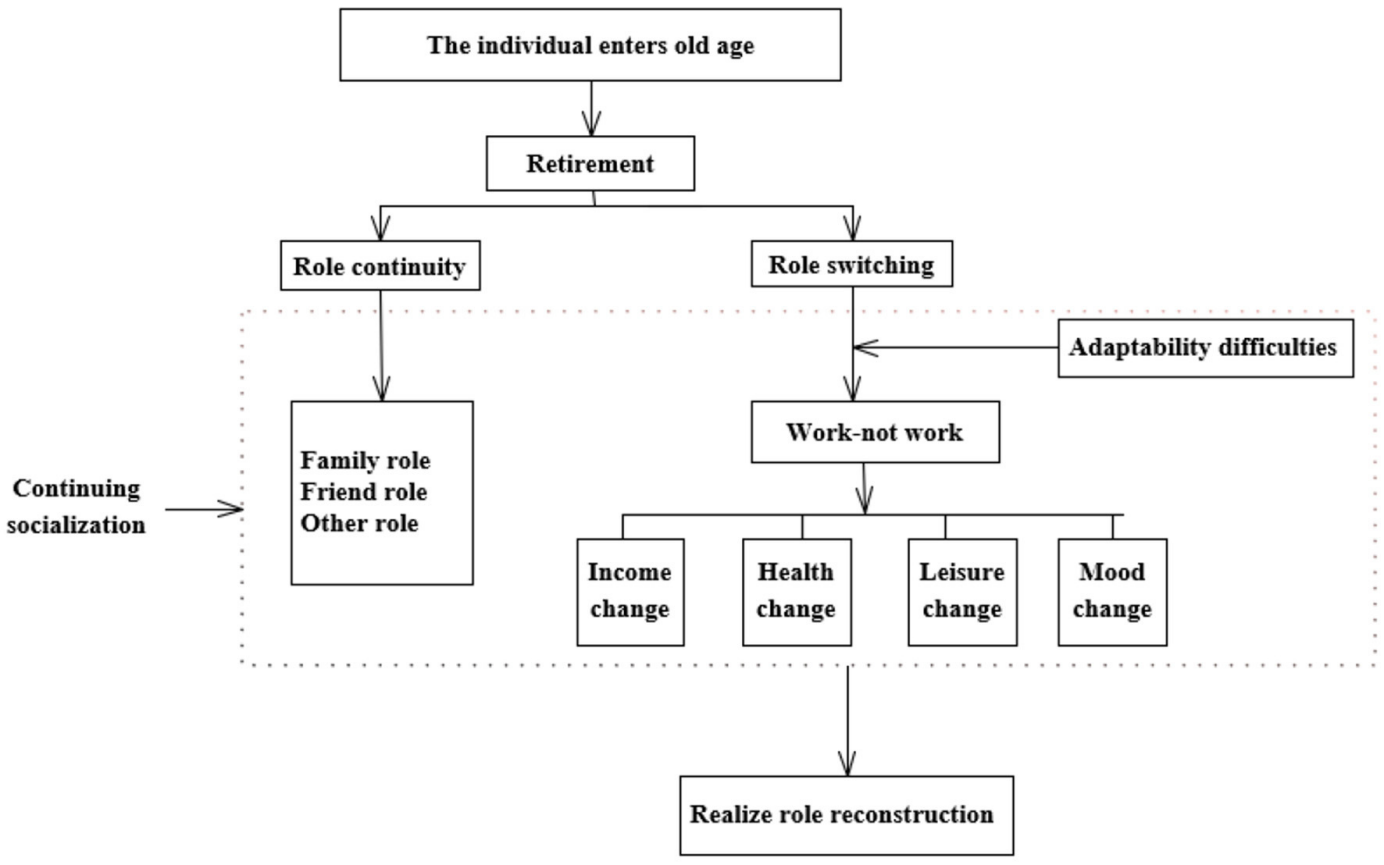
The impact of retirement.

As shown in Figure 1, individuals will face retirement when they enter old age. After retirement, some of the roles of older adults are continuous, such as the role of family and the role of friends. Another part of roles changes as individuals move from work to non–work, and these transitions are mainly caused by changes in work status. For example, income will change after retirement (26, 27) and health will improve after retirement (28–32). Social interaction activities will be significantly reduced after retirement (29). Individuals also experience significant improvements in psychological mood as a result of retirement (30). The changes in income, health, social communication, and mood caused by the change of role will affect personal utility (33), and continued socialization in old age will in turn affect happiness. This paper hypothesizes that retirement will moderate happiness through income, health, social interaction status, and mood changes to achieve role reconfiguration.

## Data

The data used in this paper comes from the data of the Chinese General Social Survey (CGSS), which started in 2003 and is China's earliest national, comprehensive, and continuous academic survey project. The CGSS system comprehensively collects data at multiple levels of society, communities, families, and individuals. After sorting out, it is found that the variables involved in this study exist in the CGSS data of 2012, 2015, and 2017, while they are missing in other years. Therefore, the survey data for these years are selected. In addition, the author has done the following processing on the selected data for 2012, 2015, and 2017: (1) In many countries in the world, women's retirement is affected by factors such as their husbands' retirement status and family characteristics, and their retirement behaviors are complex (34–36). The same is true of Chinese women's retirement behavior (37). In addition, China's retirement policy for female employees stipulates that the general female retirement age is 50 years old, the retirement age of female cadres is 55 years old, and female employees who are engaged in special types of work or are seriously ill can retire at 45 years old. In view of the complexity of women's retirement, China has formulated a retirement policy that is different from that of men, and this policy further enhances the choice of women's retirement. Some scholars have found that the complexity of Chinese women's retirement behavior does exist in reality, and many women apply for retirement before reaching the normal retirement age (38). Men have higher compliance with the mandatory policy of retirement at age 60, and are more suitable for research using the method of FRD. Therefore, this paper only retains male samples for the sake of scientific research and identification accuracy; (2). Due to historical reasons, in the 1950s, a retirement system was introduced to protect the right of urban workers. At that time, the only employer was either the government or state–owned enterprises and institutions. After economic reforms in the 1980s, the private sector and self–employed sectors entered the market. Retirement policies have been adjusted to cover urban workers in these “new” sectors, but still do not apply to rural China. As long as farmers are in good health, they usually continue to work. Therefore, in this study, we limit the analysis to urban residents (3). This paper eliminates the missing values of the main variables such as happiness, age, and retirement. At the same time, in order to better control sample selection bias and study the impact of retirement decisions on happiness, we exclude data for people older than 80 and younger than 40. Finally, 4,778 valid samples were selected, including 2876 non–retired samples and 1902 retired samples.

## Variable

### Outcome Variable

The outcome variable of this research is happiness, which specifically refers to subjective happiness, that is, people's evaluation of their own lives, including emotional and cognitive aspects (39). In the individual questionnaire, the answer to the question “In general, do you think your life is happy?” is set into five categories: “very unhappy, relatively unhappy, not happy or unhappy, relatively happy, very happy”, and they are respectively assigned a value of 1 to 5, in order to clearly distinguish whether an individual is happy or not, this study re–assigns the variables, the author defines the three categories of “very unhappy, relatively unhappy, and not happy or unhappy” as unhappiness, assigning a value of 0. Comparatively, the two categories of “relatively happy and very happy” are defined as happiness, with a value of 1.

### Retirement

With reference to ZhangYi et al. we regard “retired” personnel and those who are no longer paid as retired personnel (26). The questionnaire asked, “Did you work for more than 1 h (including joining the army) in order to obtain income last week?” For this question, the option “not engaged in any work for the purpose of obtaining economic income” is assigned a value of 1 the option “paid leave, study, temporary suspension or seasonal closure, etc.” is assigned a value of 2. “Unpaid leave, study, temporary suspension or seasonal closure, etc.” is assigned a value of 3. “Yes, I work every week” is assigned a value of 4. This paper defines the three groups of people with a value of 2–4 as non–retired people and reassigned them a value of 0. Define “retirement” as retired population and assign a value of 1. Further, the questionnaire asked “What was the reason why you did not work last week?” and set 9 options of “study at school,” “loss of workability,” “non–working after graduation,” “loss of original job due to reasons of employers,” “loss of original job due to personal reasons,” “expropriation of contracted land,” “retirement,” “housework,” and “other reason.” This article excludes the option “study at school” and “others” and defines the six types of “loss of labor,” “non–working after graduation,” “loss of original job due to reasons of employers,” “loss of original job due to personal reasons,” “expropriation of contracted land,” and “housework” as the unretired population, and assigns a value of 0, This treatment is basically done in consideration of the fact that these six types of cases enjoy before and after reaching retirement age the treatment is different if the individual can enjoy disability benefits before reaching retirement age, there is no financial security for unemployment, but the annuity security from paying pension insurance will be legally enjoyed after retirement. In addition, the article removes the above six cases from the sample in the robustness test, defines unretirement more strictly, and tests the robustness of the effect of retirement on happiness.

### Normalized Age

The driving variable in this article is the retirement age, and the legal retirement age for men in my country is 60 years old. With reference to Zhang Yi et al. we define the standardized age “a” as the actual age of men minus the legal retirement age of 60 years: a is calculated as follows: a = male's age−60 years (26). The questionnaire asked,” What is your date of birth? (Record the year and month of the Gregorian calendar).” This article uses the year and month when the survey was conducted to subtract the year and month of the birth date to get the age of the respondent. and then subtract 60 from the age of the respondent to get the value of “a”.

### Control Variables

Personal happiness is a composite indicator, which is also influenced by many other factors. According to the current research, it is mainly manifested in the following aspects. Factors such as faith status, education level, whether one participates in health insurance, the number of children, and the respondent's region can directly affect older people's evaluation of happiness (40, 41). In this paper, control variables are included to improve the reliability of the regression results. The specific variable selection and definitions are shown in Table 1.

**Table 1:** Variable definition.

**Variables**	**Variable definition**
Happiness	Respondents' happiness: unhappiness = 0, happiness = 1
Retirement	Retirement status of respondents: not retired=0, retired=1
Age	Respondents' age
Belief	Religious beliefs of interviewees: Yes = 0, none = 1
Medicare	Respondent's medical insurance situation: not participating=0, participating = 1
Edu	Respondent's education level, unit: year
Child	Number of children the respondent has: 0 or 1 = 0, 2 or more = 1
Region	Region of the respondent: East = 1, Central = 2, West = 3
Income	Respondent's income, unit: ten thousand yuan
Health	Respondent's health status: 1–5, the larger the value, the healthier
Social_i	Respondents' social interactions: 1–5, the greater the value, the more frequent the interaction
Motion	Respondent's motion:1–10, the greater the value, the better the mood

Before setting up the model, this paper first conducts basic Summary statistics to show the statistical characteristics of variables. Table 2 is the descriptive statistics of the research samples, which are divided into full samples, non–retired samples, and retired samples. Among them, happiness is the outcome variable, and the average value of the full sample is 0.791, the mean of the non–retired sample is 0.762, and the mean of the retired sample is 0.835, which indicates that the overall happiness of the surveyed group is high, and the average value of happiness after retirement has increased by 0.073 compared with the pre–retirement sample, indicating that retirement may have a positive effect on happiness, and further research will be carried out below. In this paper, age is the driving variable. The overall sample average is 57.977 years old; the retired sample average is 67.028 years old, and the non–retired sample average is 51.991 years old. Among the control variables, the average years of education of the retired sample are significantly lower than that of the non–retired sample, with a difference of about 1 year; in terms of the number of children, the number of children in the retired sample is significantly higher than that in the non–retired sample, which has an average of 2 more children. Among the mechanism variables, in terms of health status, the health level of the non–retired group is 0.263 higher than the average of the retired group; in terms of income, the average annual income of the retired group is 13,26 thousand yuan lower than that of the non–retired group.

**Table 2:** Summary statistics.

**Variables**	**ALL**	**Non–Retirement**	**Retirement**
Happiness	0.791	0.762	0.835
Retire	0.398	0	1
Age	57.977	51.991	67.028
Age^2^	3476.968	2774.542	4539.101
Belief	0.917	0.911	0.927
Medicare	0.939	0.922	0.965
Child	0.396	0.305	0.533
Social_i	2.684	2.743	2.595
Region	1.566	1.63	1.468
Edu	10.789	11.159	10.231
Income	4.579	5.107	3.781
Health	3.539	3.644	3.381
Motion	0.929	0.917	0.947
Observations	4778	2876	1902

## Empirical Strategy

Our aim is to estimate the causal effect of retirement on happiness. We start with a linear model:


(1) $$\begin{array}{r} Happiness_{i}=\beta_{1}Retire_{i}+\delta X_{i}+\varepsilon_{i} \end{array}$$

*Happiness*_*i*_ represents a sense of happiness, 0 represents unhappiness, 1 represents happiness; *Retire*_*i*_ represents retirement, 0 represents not retired, 1 represents retired; *X*_*i*_ is other control variables that affect happiness; ε_*i*_ is the error term, which is an uncontrolled variable that affects happiness. β_1_ is the parameter that the author focuses on, reflecting the way and extent of the impact of retirement on happiness.

Because the main explanatory variable retirement is a binary variable, this paper further uses the binary logit regression model for regression. The equation is as :


(2) $$\begin{array}{r} \ln\left(\frac{p_{i}}{1-p_{i}}\right)=\alpha +\overset{k}{\underset{j=1}{\sum }}\beta_{ij}x_{ij}+\varepsilon_{i} \end{array}$$

Equation (2) is the binary logit regression model. The probability of whether the ith individual retires is *p*_*i*_, where *p*_*i*_ = (*y*_*i*_ = 1|*x*_*ij*_).1–*p*_*i*_ represents the probability of not retiring. α is a constant term, β_*ij*_ is the coefficient of the independent variable, reflecting the way and degree of retirement's influence on happiness. The ratio of the probability of retirement to non–retirement, *p*_*i*_/(1−*p*_*i*_) is called the Odds ratio, abbreviated as “OR,” which is positive and has no upper bound.

To identify the causal relationship between retirement and happiness, it is necessary to consider the estimation bias caused by possible reverse causality and missing variables. If happiness has an impact on retirement, reverse causality may play a role. For example, individuals with low happiness may tend to retire early; there may also be estimation biases caused by missing variables, such as unobserved influencing factors of personal life satisfaction. To deal with the above–mentioned endogenous problems and accurately identify the causal relationship between retirement and old age happiness, this article further adopts the method of Regression Discontinuity (RD) to identify causality. RD was first used by Thistlewaite and Campbell. It was not until the end of the 1990s that it attracted the attention of economists. RD can effectively identify the causal relationship between variables (42–44). The basic idea of the regression discontinuity (RD) method is that a cause variable (D) is completely determined by whether a reference variable (X) exceeds a certain breakpoint. If the reference variable (X) will affect the outcome variable (Y) at this point, then one can assume that the relationship between the two is continuous, and other factors that may affect the outcome are also continuous at the cutoff point, the jump of the result variable at the cutoff point can be said to be caused by the cause variable (D). Regression discontinuity (RD) methods are divided into two types: sharp regression dis– continuity (Sharp RD) and fuzzy regression discontinuity (Fuzzy RD). The characteristic of sharp regression discontinuity is that the probability of an individual being processed at the cutoff point *X* = *x*0 jumps from 0 to 1; but in many cases, the causal variable is often affected by factors that cannot be observed by researchers and are not completely determined by the reference variable. In this case, the fuzzy regression discontinuity method is more appropriate, that is, the probability of an individual being processed at the cutoff point *X* = *x*_0_ jumps from a to b, where 0 < *a* < *b* < 1.

**Figure 2:**
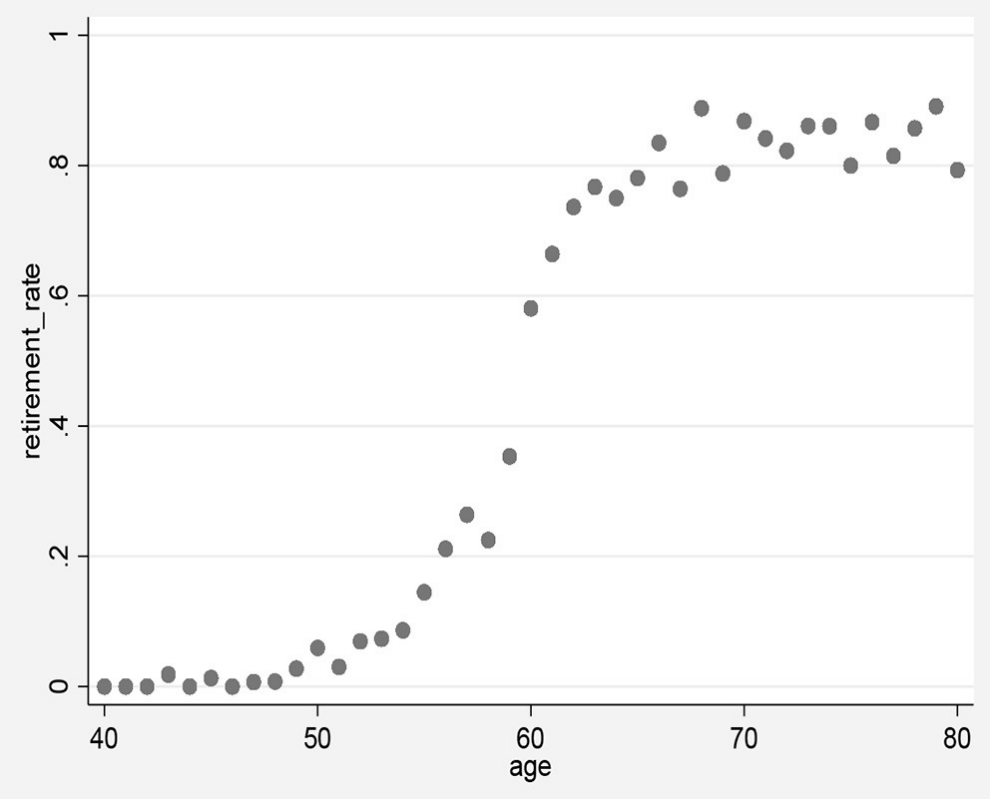
The impact of retirement.

China's retirement system fits exactly this feature. In principle, employees must retire at their statutory retirement age, but there may be special circumstances: (1) If employees' work is harmful to health, they are allowed to retire 5 years earlier than the statutory retirement age, or if the medical examination proves that they are seriously ill to continue working, they can retire early. (2) Among employees in the private sector, self–employed and temporary employees, the retirement age is not strictly enforced as in the public sector and state–owned enterprises. Therefore, people do not fully comply with the legal retirement age. A considerable number of people retire before reaching the retirement age, and a considerable number of people are still working for salaries after reaching the statutory retirement age. Therefore, it may not be directly from 0 to 1 at the breakpoint. As we will show later in this study, we do observe discontinuities in retirement rates in this age group. It can be seen from Figure 2 that for male employees, the retirement probability has a big jump around the age of 60, from about 30% before retirement to about 60% after retirement. The retirement rate at the cutoff point x = 60 is indeed not a direct jump from 0 to 1, which is more in line with the characteristics of fuzzy regression discontinuity. Therefore, this paper uses fuzzy regression discontinuity (Fuzzy RD) to process the data.

According to the theorem of Hahn et al. we can get the average treatment effect of retirement on personal happiness, the equation is as follows (45):


(3) $$\text{E}\left[\tau_{i}\text{|}X_{i}=x_{0}\right]=\frac{\underset{x↓x_{0}}{\lim}E\left[Y_{i}\text{|}X_{i}=x_{0}\right]-\underset{x↑x_{0}}{\lim}E\left[Y_{i}\text{|}X_{i}=x_{0}\right]}{\underset{x↓x_{0}}{\lim}E\left[D_{i}\text{|}X_{i}=x_{0}\right]-\underset{x↑x_{0}}{\lim}E\left[D_{i}\text{|}X_{i}=x_{0}\right]}$$

The variable *X*_*i*_ in this paper is the difference between the sample age and the cutoff point of 60 years old. The result variable is the happiness of the sample. The treatment variable *Di* is defined as whether to retire or not. If yes, then *D* = 1; if not, then *D* = 0. We estimate the difference in happiness between the retired sample and the non–retired sample based on formula (3), that is, the treatment effect.

To better identify the average treatment effect of retirement on happiness, this paper follows the idea of Hahn et al. adopts a parameter estimation method, using instrumental variables to estimate the effect of retirement on happiness (45). First, an equation for the effect of retirement on happiness is established:


(4) $$\begin{array}{r} Y_{i}=\beta_{0}+\beta_{1}R_{i}+\beta_{2}f\left(S\right)+\beta_{3}U_{i}+\varepsilon_{i} \end{array}$$

In model (4), *Y* represents personal happiness; *R*_*i*_ represents whether to retire, a value of 1 represents retirement, 0 represents not retired; *U*_*i*_ is a control variable, including belief, edu, medicare, child, and region. β_1_ represents the causal relationship between retirement and happiness, which is the average treatment effect of the FRD model *ATE*_*FRD*_. S represents the difference between age and legal retirement age. *f* (*S*) represents a higher–order polynomial containing *S*. In this paper, high–order polynomials are added to construct non-linear relations for Fuzzy regression discontinuity to prevent the variable S from being correlated with the error term to cause errors. The order of the polynomials is judged using AIC (Akaike Information Criterion) (44).

Hank and Korbmacher pointed out that retirement *Ri* is affected by many factors such as family demographic structure (46). It is obviously related to the variables omitted in a model (4), and they are all included in the interference items. This will cause endogenous problems, so the direct use of the original variable *Ri* for OLS estimation is likely to lead to estimation bias, and the use of an exogenous variable can avoid this situation. Individual retirement is highly correlated with age, and there is no ambiguity in age, which has a strong exogenous nature. Using age–related instrumental variables can accurately estimate the retirement effect. Therefore, we adopt the two–stage least squares method, that is, to find the instrumental variables for retirement in the first stage and use the model (2) to perform regression calculation on the instrumental variables in the second stage to avoid endogeneity problems. The specific method is to use possible exogenous variables to regress *Ri* to obtain the estimator of *Ri*, which is used as an instrumental variable. For this purpose, the following model is established:


(5) $$\begin{array}{r} R_{i}=\alpha_{0}+\alpha_{1}D_{i}+\alpha_{2}g\left(S\right)+\alpha_{3}V_{i}+\delta_{i} \end{array}$$

In this model, *g*(*S*) is a high–order polynomial containing *S*. Here, we use the difference between the sample individual's age and the legal retirement age as an instrumental variable, namely *D*_*i*_. When the age difference is >0, *D*_*i*_ takes the value 1, and these samples are the experimental group; When the age difference is <0, the value of *D*_*i*_ is 0, and these samples are the control group. Therefore, we can use the idea of instrumental variables combined with the breakpoint parameter estimation method (IV/RD) for analysis. The IV/RD parameter regression results in the subsequent empirical part of this article are all estimated using formula (4) and formula (5), that is, using two stage least square to obtain Fuzzy regression discontinuity estimates.

## Main Results

We first show the preliminary regression results using the linear model and the binary logit model, as shown in Table 3. Among them, models (1), (2), and (3) are linear regression results, models (4), (5), and (6) are binary logit regression results, models (1) and (3) are results without control variables, models (2) and (4) is the regression result after adding the control variables and age, and models (3) and (6) is the regression result after adding the control variables, age, and age^2^.

**Table 3:** Main results.

	**(1)**	**(2)**	**(3)**	**(4)**	**(5)**	**(6)**
		**OLS**			**Logit**
Retire	0.075^***^	0.041 ^**^	0.039^**^	0.073^***^	0.040^**^	0.040^**^
	(0.012)	(0.017)	(0.016)	(0.012)	(0.016)	(0.016)
Age		0.002^**^	−0.011^*^		0.001^*^	−0.008
		(0.001)	(0.006)		(0.001)	(0.006)
Age^2^			0.001^**^			0.000^*^
			(0.000)			(0.000)
Belief		−0.036	−0.035		−0.035^*^	−0.034^*^
		(0.022)	(0.022)		(0.020)	(0.020)
Edu		0.012^***^	0.012^***^		0.012^***^	0.012^***^
		(0.002)	(0.002)		(0.002)	(0.002)
Medicare		0.100^***^	0.101^***^		0.125^***^	0.126^***^
		(0.021)	(0.021)		(0.028)	(0.028)
Child		0.073^***^	0.067^***^		0.071^***^	0.067^***^
		(0.014)	(0.014)		(0.014)	(0.014)
Region		−0.005	−0.005		−0.006	−0.006
		(0.008)	(0.008)		(0.008)	(0.008)
*N*	4778	4778	4778	4778	4778	4778

^***^, ^**^, ^*^ indicate significance at the level of 1, 5, and 10% respectively; the numbers in parentheses are the standard errors. The logit regression reports the marginal effect.

After the estimation of the Fuzzy regression discontinuity model is completed, the model needs to be tested as follows to ensure the robustness of the estimation results. First of all, according to the research content of Lee and Lemieux, the breakpoint regression design needs to meet the local randomization assumption (29), that is, the reference variable can't be accurately controlled by individuals, otherwise the Fuzzy regression discontinuity will fail. Therefore, according to the test method proposed by McCrary, this article checks whether the sample age distribution is continuous at the cutoff point to test whether it meets the hypothesis of local randomization (47); Secondly, if the distribution of covariates also “jumps” at the cutoff point breakpoint, it cannot prove that the “jump” of the outcome variable at the cutoff point is due to the influence of the cause variable. The fuzzy regression discontinuity design will also lose its effect, so we need to test the continuity of the covariate at the cutoff point; Next, we will select different sample intervals for regression to further test the robustness of the results. Then, we do the analysis with income, health, social interaction, and mood as the mechanism variables. Finally, this paper tests the heterogeneity of changes in post–retirement happiness across groups with different education levels and number of children.

### Basic Regression Results

It can be seen from Table 3 that the estimated results of OLS regression and binary logit regression are consistent in terms of significance, which shows that retirement significantly improves happiness. As far as the control variables are concerned, the higher the education level, the happier; Those who participated in medical insurance were significantly happier. Those with more children are significantly happier than those with fewer children. These variables are all significant at the 0.01 level. In addition, people without religious beliefs are happier after retirement. Geographically, the closer to the east, the happier but this is not statistically significant.

In order to make the estimation results more accurate and effective, we use Fuzzy regression discontinuity design to estimate again to solve the possible endogenous problems and sample selection bias problems. This paper refers to the research of some scholars and uses the rdbwselect method to estimate the optimal bandwidth 4, and the standardized age a and its square *a*^2^ are respectively put into the model for regression (48–51). The specific regression results are shown in Table 4.

**Table 4:** FRD estimation results.

	**(1)**	**(2)**
	**bandwidth 4**	
Retire	0.228^***^	0.225^***^
	(0.052)	(0.052)
a	0.086^***^	0.086^***^
	(0.005)	(0.005)
		−0.003
		(0.002)
Covariates	Yes	Yes
Observations	1290	1290

^***^ indicate significance at the levels of 1%; in the parentheses are the standard errors.

The research results in Table 4 show that using Fuzzy regression discontinuity design can still draw conclusions that retirement improves happiness. When the driving variable of the model is only standardized age, the coefficient is 0.228, which is significant at the level of 0.01, which further validates the conclusions in Table 3. At the same time, in the first stage of regression, the driving variable a is significant at the level of 0.01, and the older the age the greater the possibility of retirement, which is consistent with the actual situation, indicating that the model settings are in line with reality. When the driving variable adds *a*^2^, the coefficient is 0.225, which is significant at the level of 0.01, but *a*^2^ is not significant, indicating that the image doesn't fit well, and the relationship between age and happiness is linear instead of an inverted U– shaped relationship. This is for reference only. This result is consistent with the study of Jokela et al. (20).

### Specifications Checks

To ensure the credibility of the regression results, the control variables need to be tested, including belief, edu, medicine, child, and region. If the density distribution functions of these control variables also have “jumps” at the breakpoints, then the “jumps” of the outcome variables cannot be fully explained by the “jumps” of the processed variables, and the causal inference will lose its effectiveness. We use the idea of Fuzzy regression discontinuity to replace the original outcome variables with these covariates and obtain the change values of these control variables in Table 5 before and after the cutoff point. From the results in the table, it can be seen that, except for medical insurance participation, these variables did not change significantly before and after the cut–off point, which further verified the credibility of the research conclusions. China's medical insurance system and retirement system are two independent systems retirement time is not affected by medical insurance participation status. When medical insurance participation changes significantly before and after the retirement threshold, controlling for medical insurance participation may lead to an underestimation of results, that is, retirement actually has a greater impact on well–being. It has no effect on our empirical conclusion. After the retirement age threshold, the participation rate of individual medical insurance increases significantly at the level of 5%, which may be due to the following reasons. On the one hand, urban and rural residents' medical insurance implements voluntary insurance, There must be an adverse selection problem in insurance. Only those who think it is cost–effective to participate in the insurance, otherwise they will not participate in the insurance. The older you are, the more health problems you may face and the need for medical insurance, so the older you are, the more likely you are to participate in the insurance. On the other hand, although the government has invested a lot of financial subsidies in medical insurance for urban and rural residents, for some urban and rural households, the cost of participating in the insurance is still a lot of expenses, and some families are unwilling to participate in the insurance. When the older person reach the retirement age, they can buy medical insurance if they get a pension. So there are more people participating in medical insurance after retirement than before retirement. In addition, in terms of the reimbursement ratio of medical insurance, some regions stipulate that the reimbursement standard for those who have reached the retirement age is higher than that for those who have not reached the retirement age. Some regions stipulate that the older the age, the higher the reimbursement rate. It may also lead to individuals being more likely to enroll in health insurance when they reach retirement age.

**Table 5:** Continuity test of control variables.

**y**	**Coef**.	**Std.Err**.	**z**	**P>z**	**[95%Conf**.	**Interval]**
Belief	−0.018	0.048	−0.380	0.704	−0.113	0.076
Edu	0.623	0.623	1.000	0.317	−0.598	1.845
Medicare	0.110	0.050	2.200	0.028	0.012	0.208
Child	0.096	0.087	1.100	0.272	−0.075	0.266
Region	−0.154	0.153	−1.010	0.312	−0.454	0.145
Lwald	−0.020	0.086	−0.230	0.817	−0.189	0.149

**Figure 3:**
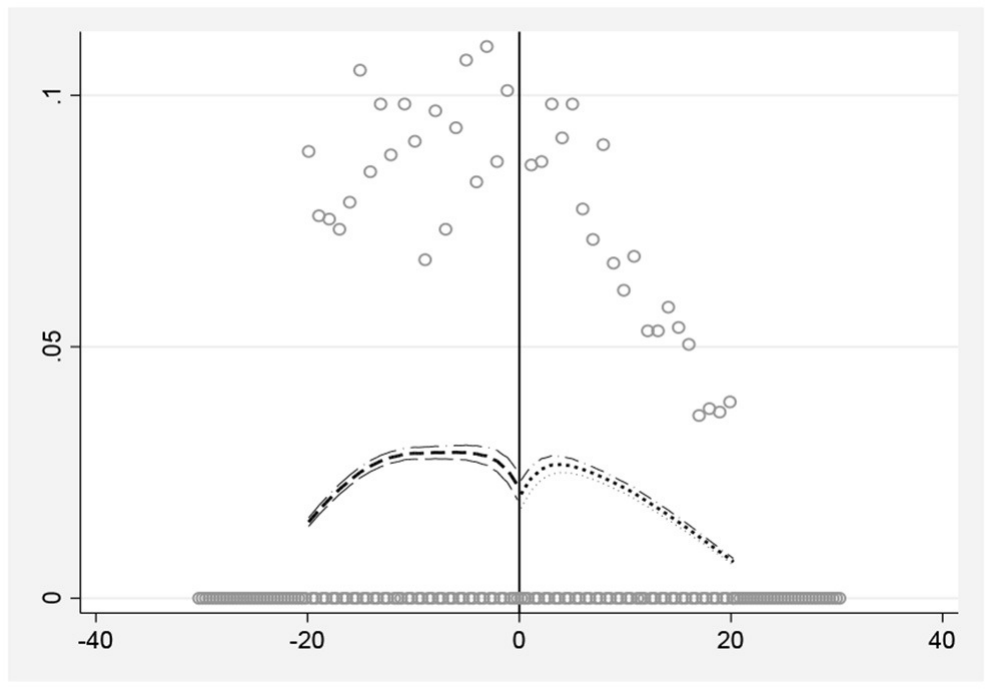
The impact of retirement on happiness.

When performing Fuzzy regression discontinuity design, it is necessary to check whether there is endogenous grouping. If the individual knows the grouping rules in advance and can fully control the grouping variables through their own efforts, they can choose to enter the experimental group or the control group by themselves, resulting in endogenous grouping rather than random grouping near the cutoff point and causing the Fuzzy regression discontinuity design to fail. Based on the calculation method of McCrary, this paper examines whether there is precise control of the assigned variable by examining the continuity of the density function of the assigned variable at the cutoff point (47). Figure 3 shows the density distribution diagram of the time between the age of the sample and the cutoff point. As can be seen in Figure 1, the density function curves all nearly overlap at the breakpoints without significant jumps. The density difference before and after the cutoff point is 0.084, and the standard error is 0.099, indicating that the age distribution of the sample individuals is continuous and smooth, that is, the individuals have not precisely manipulated the grouping variables.

### Test of the “Jump” of the Explained Variable at the Cutoff Point

**Figure 4:**
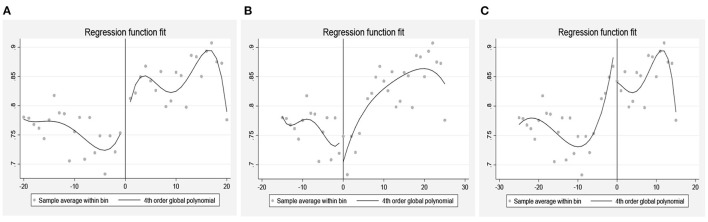
**(A)** The effect of retirement on happiness when 60 is the retirement age. **(B)** The effect of retirement on happiness when 55 is the retirement age. **(C)** The effect of retirement on happiness when 65 is the retirement age.

Figure 4A shows that there is indeed a significant jump in happiness around the age of 60. Figures 4B,C are the placebo tests on the assumption that the retirement age of the older person is 55 and 65 years old respectively. As can be seen from the figure, there is no significant change in happiness near the age of 55 and 65.

### Robustness Check

To test the robustness of the results, this paper selects 50% and 200% of the optimal bandwidth, namely bandwidth 2 and bandwidth 8, to investigate the impact of retirement on individual happiness. The results are shown in Table 6. As can be seen from the table, the coefficient gradually decreases with the increasing sample size. That's because the larger the sample range, the weaker the impact of retirement on happiness. This is determined by the intrinsic characteristics of the fuzzy regression discontinuous design model. The coefficient on retirement is significant at the 0.01 level, the standardized age term is always significant, and the standardized age–squared term is significant at broadband 8 insignificant at broadband 4. This indicates that the model is robust and less dependent on bandwidth.

**Table 6:** FRD estimation results using different bandwidth.

	**(1)**	**(2)**	**(3)**	**(4)**
	**bandwidth 2**		**bandwidth 8**	
Retire	0.175^**^	0.169^*^	0.149^***^	0.149^***^
	(0.087)	(0.087)	(0.030)	(0.030)
a	−0.022^**^	0.104^***^	0.062^***^	0.062^***^
	(0.010)	(0.007)	(0.002)	(0.002)
		0.004		−0.001^**^
		(0.005)		(0.000)
Covariates	Yes	Yes	Yes	Yes
Observations	722	722	2342	2342

^***^, ^**^, ^*^ indicate significance at the levels of 1, 5, and 10% respectively; in the parentheses are the standard errors.

In the previous sample, the non–retired sample included those who studied at school, lost the ability to work, did not work after graduation, lost their original job due to their employer, lost their original job due to personal reasons, did housework, and contracted land is expropriated. The above–mentioned samples are removed here, a more rigorous definition of retirement and non–retirement to do robustness tests. the optimal bandwidth 4 was estimated using the rdbwselect method, and the standardized age a and its square a^2^ are respectively put into the model for regression. To test the robustness of the results, 50% and 200% of the optimal bandwidth, namely bandwidth 2 and bandwidth 8 were selected to investigate the impact of retirement on individual happiness. It is clear from the Table 7 that retirement can still significantly improve happiness after adopting a stricter definition of retirement.

**Table 7:** The impact of retirement (redefined) on happiness.

	**(1)**	**(2)**	**(3)**	**(4)**	**(5)**	**(6)**
	**bandwidth 4**		**bandwidth 2**		**bandwidth 8**	
Retire	0.192^***^	0.187^***^	0.124	0.123	0.103^***^	0.105^***^
	(0.054)	(0.055)	(0.091)	(0.091)	(0.030)	(0.030)
a	0.085^***^	0.085^***^	0.135^***^	0.136^***^	0.063^***^	0.063^***^
	(0.005)	(0.005)	(0.013)	(0.013)	(0.002)	(0.002)
		−0.004^*^		−0.020^*^		−0.001^***^
		(0.002)		(0.011)		(0.000)
Covariates	Yes	Yes	Yes	Yes	Yes	Yes
Observations	1152	1152	641	641	2092	2092

^***^, ^*^ indicate significance at the levels of 1 and 10% respectively; in the parentheses are the standard errors.

### Alternative Estimation Methods

In order to test the robustness of the results of this paper, the sample whose age is exactly 60 years old is deleted in this paper, the products of a, *a*^2^, *a*^3^ and retirement are put into the model separately, that is, using the method of local polynomial regression:


(6) $$\begin{array}{r} Y_{i}=f\left(a\right)+\beta_{1}\;\times \;post60_{i}\;+\;f\left(a\right)\;\times \;post60_{i}+\varepsilon_{i} \end{array}$$

As can be seen from Table 8, retirement still significantly improves happiness, and its impact on happiness is also Robust.

**Table 8:** The impact of retirement on happiness.

	**(1)**	**(2)**	**(3)**
Retire	0.0516^*^	0.0657^*^	0.0596^*^
	(0.0169)	(0.0180)	(0.0217)
a	0.00431^*^	0.00629^*^	0.00603^*^
	(0.0009)	(0.0010)	(0.0020)
Retire x a	−0.00255	−0.00254	−0.00158
	(0.0016)	(0.0026)	(0.0032)
a^2^		0.000218^*^	0.000230^*^
		(0.0001)	(0.0001)
Retire x a^2^		−0.000354^**^	−0.000167
		(0.0002)	(0.0003)
a^3^			0.00000135
			(0.0000)
retire x a^3^			−0.0000141
			(0.0000)
Covariates	Yes	Yes	Yes
Observations	4592	4592	4592

^**^, ^*^ indicate significance at the levels of 5 and 10% respectively; in the parentheses are the standard errors.

### Mechanisms Analysis

How does retirement affect personal happiness? We try to identify the specific mechanisms of the impact of retirement on happiness to enrich empirical studies in China, as well as to find possible compensation options for future welfare losses resulting from the implementation of delayed retirement policies in China. Based on role theory and continuous socialization theory, we argue that retirement may cause different changes in happiness through changes in residents' income, health status, social interaction activities, and emotional status, so we test these four aspects. The empirical method used is:


(7) $$\begin{array}{l} happiness_{i}=\alpha_{0}+\alpha_{1}retire_{i}+\alpha_{2}mediator_{i}+\\ \;\alpha_{3}retire_{i}\;x\;mediator_{i}+\alpha_{4}X_{i}+\;\varepsilon_{i} \end{array}$$

As shown in Table 9, health and social interactions affect the increase in happiness in retirement. The better the health status, the smaller the effect of retirement on happiness. The more social interactions, the smaller the effect of retirement on happiness. Income and emotional status do not affect the relationship between retirement and happiness.

**Table 9:** Analysis of mediation effect.

	**(1)**		**(2)**		**(3)**		**(4)**
Retire	0.158^***^	retire	0.108^***^	retire	0.050^***^	retire	0.058
	(0.048)		(0.034)		(0.018)		(0.060)
Health	0.107^***^	social_i	0.044^***^	income	0.004^***^	motion	0.317^***^
	(0.008)		(0.007)		(0.001)		(0.033)
h x r	−0.036^***^	s x r	−0.026^**^	i x r	−0.002	m x r	−0.033
	(0.012)		(0.011)		(0.002)		(0.060)
Covariates	Yes	Yes	Yes	Yes	Yes	Yes	Yes
N	4778	4778	4778	4778	4778	4778	4778

^***^, ^**^ indicate significance at the levels of 1 and 5% respectively; in the parentheses are the standard errors.

### Heterogeneity Analysis

In order to find out how retirement affects personal happiness, and whether there is heterogeneity in the happiness of different groups after retirement, we will analyze the heterogeneity in groups based on education, and the number of children. Enrich China's experience and research, and at the same time find possible remedies for the loss of welfare equity caused by the implementation of the delayed retirement policy in China in the future.

From the columns (1)–(2) of Table 10, it can be seen that the happiness of the group with an education level below university is greater than that of the group with an education level and above, and there are significant differences between groups. It can be seen from the columns (3)–(4) of Table 10 that a group with multiple children is happier after retirement than a group with a single child.

**Table 10:** Analysis of the impact of retirement on the happiness of different groups.

	**(1)**	**(2)**	**(3)**	**(4)**
	**edu**		**child**	
	**Below**	**University**	**One**	**More**
	**university**	**and above**		**than one**
Retire	0.095^***^	0.028	0.042^*^	0.144^***^
	(0.023)	(0.032)	(0.025)	(0.029)
Empirical p–value	0.063	0.003
Covariates	Yes	Yes	Yes	Yes
Observations	3630	1148	2888	1890

^***^, ^*^ indicate significance at the levels of 1 and 10% respectively; in the parentheses are the standard errors.

## Discussion

This paper explores whether and to what extent the retirement system affects the happiness of urban residents, and the results show that retirement will significantly improve the happiness of Chinese people. This is the same as some research conclusions in developed countries such as the United Kingdom, the United States, and Australia (19–21). The research in this paper further expands the boundaries of the impact of retirement on happiness, and has general reference significance for developing countries.

According to our model and empirical test results of the impact of retirement on happiness, the changes in happiness among groups are heterogeneous after retirement. Health and social interactions influence increases in retirement happiness. The better your health, the less impact retirement has on happiness. The more social interaction, the less impact retirement has on happiness. This may be due to the fact that retirement can free a person from social pressures at work, increase leisure time, develop hobbies, improve relationships with family members, enhance intimacy, and thus increase happiness. Jokela et al. (20) also found that came to the same conclusion (20). The implication for the policy is that the delayed retirement policy can be targeted to publicize different groups, and firstly publicize the groups whose welfare is less damaged, such as those with good health status and more social activities, so as to reduce the resistance of the policy.

The improvement of the post–retirement happiness of the group with the education level below the university is significantly greater than that of the group with the education level of the university and above. This may be because the positions in the group below the university are lower than those with a university degree, and the work is relatively boring and tiring. The family utility and leisure utility of middle–aged and older people are greater than the income utility brought by work, so they are happier after retirement. A group with multiple children experienced a greater increase in happiness after retirement than a group with one child. This may reflect the tradition of more children and more happiness. Older adults with more children have more family ties and are happier in retirement. The implication for the policy is that the delayed retirement policy may affect the existing group welfare status, causing greater welfare losses for groups with low education levels and many children, which may require the introduction of relevant supporting measures to narrow the group welfare gap.

It should be noted that a limitation of our research was the exclusion of female retirees. Because in traditional Chinese culture, men are in charge of the outside world and women are in charge of the home, women may give up work at any time due to family and other factors, and women's retirement behavior is more complicated. China's retirement policy for women has further enhanced the selectivity of women's retirement time. In traditional cultures, women are responsible for caring for grandchildren after retirement, complicating changes in women's post–retirement happiness. Further research is needed on the issue of Chinese women's retirement and the impact of Chinese women's retirement on happiness.

## Conclusion

With the advent of the aging wave, people pay much attention to retirement policies. In order to enhance the sustainability of pensions and reduce financial pressure, the Chinese government wants to introduce a delayed retirement policy, which is bound to affect the welfare of the older person. In this context, this paper explores whether the retirement system will affect the happiness of residents and the specific extent of the impact, which is related to public decisions such as whether to launch delayed retirement, when to launch it, to whom, and how to launch it. Will retirement really make people happier? To answer the question of whether people's happiness will increase after retirement, this paper uses 2012, 2015, and 2017 China General Social Survey (CGSS) data to conduct empirical research through three methods: OLS, binary Logit, and Fuzzy regression discontinuity design. The results all show that retirement will significantly improve the happiness of people. By testing the continuity of reference variables and covariates at breakpoints and more rigorous sub–sample estimation results, the author found that the above conclusions are still robust. Retirement significantly increases the happiness of older adults, which is consistent with public opposition to delayed retirement. Some research also found that delaying retirement can harm the well–being of residents, such as affecting family care (52). Although delaying retirement can reduce the financial burden of China's pension system, it can have a negative impact on the happiness of the older person. The article further analyzes the heterogeneity of the research and found that retirement brings more happiness to people who have a college degree or less, and have multiple children. The better the health status, the smaller the effect of retirement on happiness. The more social interactions, the smaller the effect of retirement on happiness. It is important to stress that our findings apply only to male retirees, not all retirees.

In response to the empirical results obtained in this paper, this paper suggests that when implementing a delayed retirement policy, publicity should be strengthened for groups with greater welfare impairment and relevant welfare impairment compensation policy measures should be introduced. Considering the many reasons why people oppose the delayed retirement policy, such as the increase in family care needs, unfairness of the pension system, crowding out of employment, and pension wealth problems, the government needs to formulate reasonable supporting policies to solve people's worries in the process of implementing the delayed retirement.

## Data Availability Statement

Publicly available datasets were analyzed in this study. This data can be found here: http://cgss.ruc.edu.cn.

## Author Contributions

Material preparation, data collection, and analysis were performed by YZ and YT. The first draft of the manuscript was written by AZ. All authors commented on previous versions of the manuscript. All authors read and approved the final manuscript and contributed to the study conception and design.

## Funding

This research was funded by the National Natural Science Foundation of China, Project No. 72174114 and the title of the project is Research on the Improvement of Old-age Security System in the Economy with Rapid Human Capital Progress.

## Conflict of Interest

The authors declare that the research was conducted in the absence of any commercial or financial relationships that could be construed as a potential conflict of interest.

## Publisher's Note

All claims expressed in this article are solely those of the authors and do not necessarily represent those of their affiliated organizations, or those of the publisher, the editors and the reviewers. Any product that may be evaluated in this article, or claim that may be made by its manufacturer, is not guaranteed or endorsed by the publisher.
